# {4,4′,6,6′-Tetra­iodo-2,2′-[2,2-dimethyl­propane-1,3-diylbis(nitrilo­methanylyl­idene)]diphenolato}copper(II)

**DOI:** 10.1107/S1600536812020387

**Published:** 2012-05-12

**Authors:** Hadi Kargar, Reza Kia, Tayebeh Shakarami, Muhammad Nawaz Tahir

**Affiliations:** a Department of Chemistry, Payame Noor University, PO Box 19395-3697 Tehran, I. R. IRAN; b Department of Chemistry, Science and Research Branch, Islamic Azad University, Tehran, Iran; c Department of Physics, University of Sargodha, Punjab, Pakistan

## Abstract

In the title compound, [Cu(C_19_H_16_I_4_N_2_O_2_)], the Cu^II^ atom and the substituted C atom of the diamine segment lie on a crystallographic twofold rotation axis. The geometry around the Cu^II^ atom is distorted square-planar, which is supported by the N_2_O_2_ donor atoms of the coordinated Schiff base. The dihedral angle between the symmetry-related substituted benzene rings is 29.40 (19)°. In the crystal, a short I⋯I [3.8766 (6) Å] contact is present and links neighbouring mol­ecules into chains propagating along the *a* axis.

## Related literature

For applications of Schiff base ligands in coordination chemistry, see: Granovski *et al.* (1993 ▶); Blower (1998 ▶). For a related structure, see: Kargar *et al.* (2012 ▶). For standard values of bond lengths, see: Allen *et al.* (1987 ▶). For van der Waals radii, see: Bondi (1964 ▶).
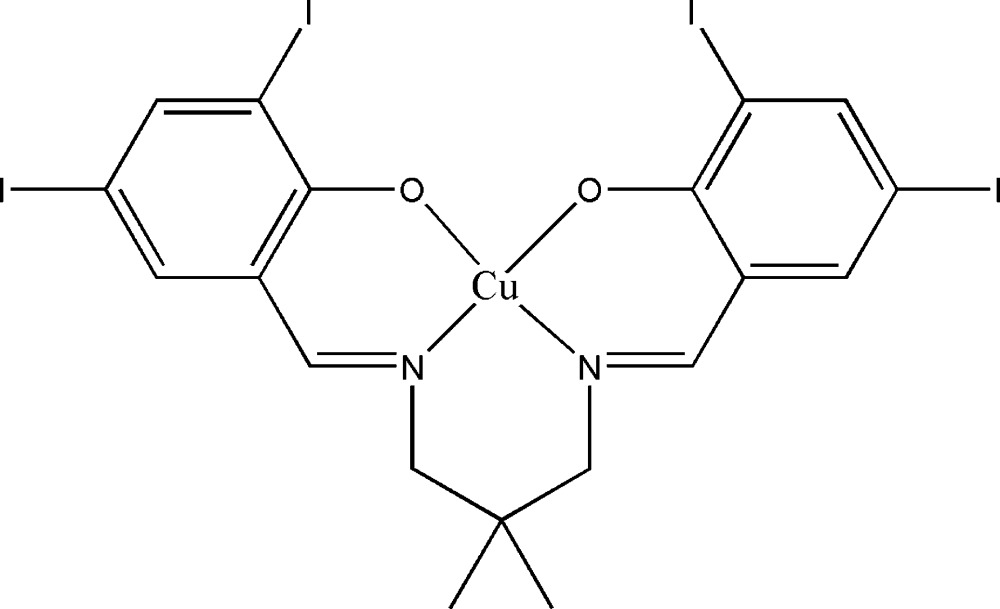


## Experimental

### 

#### Crystal data

[Cu(C_19_H_16_I_4_N_2_O_2_)]
*M* *_r_* = 875.48
Orthorhombic, 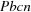

*a* = 16.9336 (10) Å
*b* = 15.9602 (12) Å
*c* = 8.7041 (5) Å
*V* = 2352.4 (3) Å^3^
*Z* = 4
Mo *K*α radiation
μ = 6.20 mm^−1^
*T* = 296 K
0.21 × 0.12 × 0.08 mm

#### Data collection

Bruker SMART APEXII CCD area-detector diffractometer
Absorption correction: multi-scan (*SADABS*; Bruker, 2005 ▶) *T*_min_ = 0.269, *T*_max_ = 0.551
9807 measured reflections
2321 independent reflections
1791 reflections with *I* > 2σ(*I*)
*R*_int_ = 0.030

#### Refinement

*R*[*F* ^2^ > 2σ(*F* ^2^)] = 0.026
*wR*(*F* ^2^) = 0.059
*S* = 1.04
2321 reflections
129 parameters
H-atom parameters constrained
Δρ_max_ = 0.94 e Å^−3^
Δρ_min_ = −0.67 e Å^−3^

### 

Data collection: *APEX2* (Bruker, 2005 ▶); cell refinement: *SAINT* (Bruker, 2005 ▶); data reduction: *SAINT*; program(s) used to solve structure: *SHELXS97* (Sheldrick, 2008 ▶); program(s) used to refine structure: *SHELXL97* (Sheldrick, 2008 ▶); molecular graphics: *SHELXTL* (Sheldrick, 2008 ▶); software used to prepare material for publication: *SHELXTL* and *PLATON* (Spek, 2009 ▶).

## Supplementary Material

Crystal structure: contains datablock(s) global, I. DOI: 10.1107/S1600536812020387/su2420sup1.cif

Structure factors: contains datablock(s) I. DOI: 10.1107/S1600536812020387/su2420Isup2.hkl

Additional supplementary materials:  crystallographic information; 3D view; checkCIF report

## Figures and Tables

**Fig. 1.**
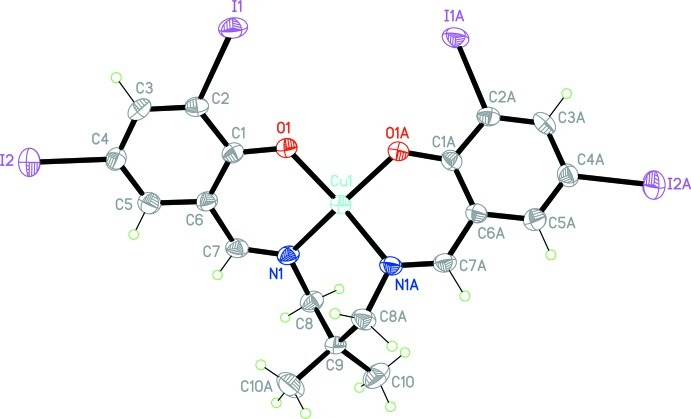
A view of the molecular structure of the title compound, showing 40% probability displacement ellipsoids and the atomic numbering [symmetry code: (A) = -x, y, -z+1/2].

**Fig. 2.**
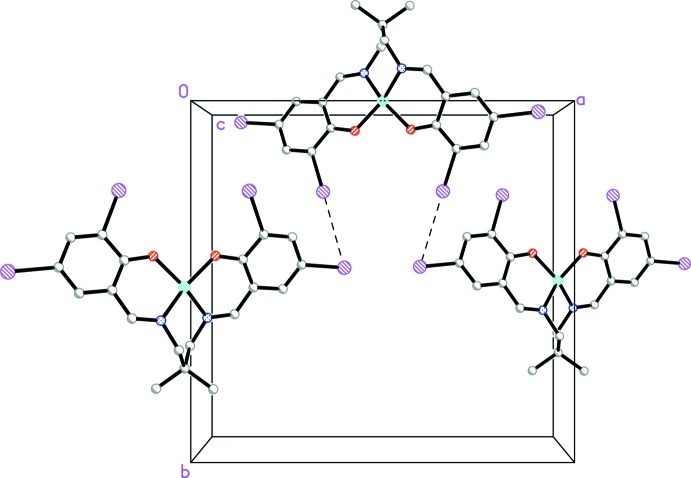
The crystal packing of the title compound viewed along the c-axis, showing how the molecules are linked via the intermolecular I···I interactions (dashed lines) to form chains along the a axis [the H atoms have been omitted for clarity].

## supplementary crystallographic information

### Comment

Schiff base complexes are one of the most important stereochemical models in transition metal coordination chemistry, owing to their ease of preparation and structural variations (Granovski *et al.*, 1993; Blower, 1998).

The asymmetric unit of the title compound, Fig. 1, comprises half of a tetradentate Schiff base ligand. Atoms Cu1 and C9 lie on a crystallographic two-fold rotation axis. The geometry around the Cu^II^ atom is distorted square-planar which is supported by the N_2_O_2_ donor atoms of the coordinated Schiff base. The dihedral angle between the symmetry-related substituted benzene rings is 29.40 (19)°. The bond lengths (Allen *et al.*, 1987) and angles are within the normal ranges and are comparable to those reported for a related structure (Kargar *et al.*, 2012).

In the crystal, a short I···I [3.8766 (6) Å] contact is present, which is shorter than the sum of the van der Waals radii of I atoms [3.96 Å; Bondi, 1964]. It links neighbouring molecules along the *a* axis forming chains.

### Experimental

The title compound was synthesized by adding 3,5-diiodo-salicylaldehyde-2,2-dimethyl-1,3-propanediamine (2 mmol) to a solution of CuCl_2_. 4H_2_O (2.1 mmol) in ethanol (30 ml). The mixture was refluxed with stirring for 30 min. The resultant solution was filtered. Red single crystals of the title compound, suitable for *X*-ray structure analysis, were obtained from ethanol by slow evaporation of the solvent at room temperature over several days.

### Refinement

The H-atoms were included in calculated positions and treated as riding atoms: C—H = 0.93, 0.96 and 0.97 Å for CH, CH_3_ and CH_2_ H-atoms, respectively, with U_iso_(H) = k x U_eq_(C), where k = 1.5 for CH_3_ H-atoms, and = 1.2 for other H-atoms.

### Crystal data

**Table:**

[Cu(C_19_H_16_I_4_N_2_O_2_)]	*F*(000) = 1604
*M**_r_* = 875.48	*D*_x_ = 2.472 Mg m^−^^3^
Orthorhombic, *P**b**c**n*	Mo *K*α radiation, λ = 0.71073 Å
Hall symbol: -P 2n 2ab	Cell parameters from 1126 reflections
*a* = 16.9336 (10) Å	θ = 2.5–27.5°
*b* = 15.9602 (12) Å	µ = 6.20 mm^−^^1^
*c* = 8.7041 (5) Å	*T* = 296 K
*V* = 2352.4 (3) Å^3^	Block, red
*Z* = 4	0.21 × 0.12 × 0.08 mm

### Data collection

**Table:**

Bruker SMART APEXII CCD area-detector diffractometer	2321 independent reflections
Radiation source: fine-focus sealed tube	1791 reflections with *I* > 2σ(*I*)
Graphite monochromator	*R*_int_ = 0.030
φ and ω scans	θ_max_ = 26.0°, θ_min_ = 2.4°
Absorption correction: multi-scan (*SADABS*; Bruker, 2005)	*h* = −20→20
*T*_min_ = 0.269, *T*_max_ = 0.551	*k* = −13→19
9807 measured reflections	*l* = −9→10

### Refinement

**Table:**

Refinement on *F*^2^	Primary atom site location: structure-invariant direct methods
Least-squares matrix: full	Secondary atom site location: difference Fourier map
*R*[*F*^2^ > 2σ(*F*^2^)] = 0.026	Hydrogen site location: inferred from neighbouring sites
*wR*(*F*^2^) = 0.059	H-atom parameters constrained
*S* = 1.04	*w* = 1/[σ^2^(*F*_o_^2^) + (0.021*P*)^2^ + 2.4697*P*] where *P* = (*F*_o_^2^ + 2*F*_c_^2^)/3
2321 reflections	(Δ/σ)_max_ = 0.001
129 parameters	Δρ_max_ = 0.94 e Å^−^^3^
0 restraints	Δρ_min_ = −0.67 e Å^−^^3^

### Special details

**Table:**

Geometry. All esds (except the esd in the dihedral angle between two l.s. planes) are estimated using the full covariance matrix. The cell esds are taken into account individually in the estimation of esds in distances, angles and torsion angles; correlations between esds in cell parameters are only used when they are defined by crystal symmetry. An approximate (isotropic) treatment of cell esds is used for estimating esds involving l.s. planes.
Refinement. Refinement of F^2^ against ALL reflections. The weighted R-factor wR and goodness of fit S are based on F^2^, conventional R-factors R are based on F, with F set to zero for negative F^2^. The threshold expression of F^2^ > 2sigma(F^2^) is used only for calculating R-factors(gt) etc. and is not relevant to the choice of reflections for refinement. R-factors based on F^2^ are statistically about twice as large as those based on F, and R- factors based on ALL data will be even larger.

### Fractional atomic coordinates and isotropic or equivalent isotropic displacement parameters (Å^2^)

**Table:**

	*x*	*y*	*z*	*U*_iso_*/*U*_eq_	
I1	0.16096 (2)	0.747572 (19)	0.30639 (4)	0.04900 (11)	
I2	0.39828 (2)	0.53650 (3)	0.63814 (5)	0.06704 (14)	
Cu1	0.0000	0.48949 (5)	0.2500	0.03357 (18)	
O1	0.07523 (17)	0.57300 (18)	0.3038 (3)	0.0384 (7)	
N1	0.0505 (2)	0.4038 (2)	0.3745 (4)	0.0348 (8)	
C1	0.1415 (2)	0.5623 (3)	0.3769 (4)	0.0332 (10)	
C2	0.1945 (3)	0.6301 (3)	0.3943 (4)	0.0350 (10)	
C3	0.2653 (2)	0.6228 (3)	0.4678 (4)	0.0384 (11)	
H3	0.2985	0.6691	0.4758	0.046*	
C4	0.2882 (2)	0.5464 (3)	0.5306 (5)	0.0394 (10)	
C5	0.2393 (3)	0.4793 (3)	0.5191 (5)	0.0417 (11)	
H5	0.2547	0.4283	0.5613	0.050*	
C6	0.1657 (2)	0.4854 (3)	0.4444 (5)	0.0348 (10)	
C7	0.1168 (2)	0.4116 (3)	0.4443 (5)	0.0364 (10)	
H7	0.1349	0.3656	0.4997	0.044*	
C8	0.0045 (3)	0.3268 (3)	0.3955 (4)	0.0401 (10)	
H8A	−0.0486	0.3416	0.4270	0.048*	
H8B	0.0281	0.2941	0.4776	0.048*	
C9	0.0000	0.2724 (4)	0.2500	0.0369 (14)	
C10	−0.0730 (3)	0.2171 (3)	0.2632 (6)	0.0599 (14)	
H10A	−0.0709	0.1862	0.3578	0.090*	
H10B	−0.0745	0.1787	0.1783	0.090*	
H10C	−0.1195	0.2515	0.2618	0.090*	

### Atomic displacement parameters (Å^2^)

**Table:**

	*U*^11^	*U*^22^	*U*^33^	*U*^12^	*U*^13^	*U*^23^
I1	0.0663 (2)	0.03097 (18)	0.04979 (18)	−0.00876 (16)	−0.00490 (15)	0.00638 (14)
I2	0.04541 (19)	0.0625 (3)	0.0932 (3)	0.00148 (19)	−0.02547 (19)	−0.0029 (2)
Cu1	0.0359 (4)	0.0272 (4)	0.0376 (4)	0.000	−0.0041 (3)	0.000
O1	0.0396 (16)	0.0278 (16)	0.0478 (17)	−0.0031 (14)	−0.0093 (14)	0.0029 (14)
N1	0.041 (2)	0.027 (2)	0.0359 (18)	−0.0058 (17)	−0.0016 (16)	0.0042 (16)
C1	0.037 (2)	0.032 (2)	0.031 (2)	−0.003 (2)	−0.0005 (18)	−0.0022 (19)
C2	0.042 (2)	0.028 (2)	0.035 (2)	−0.004 (2)	0.0031 (19)	0.0039 (19)
C3	0.038 (2)	0.037 (3)	0.041 (2)	−0.012 (2)	0.0025 (19)	−0.007 (2)
C4	0.033 (2)	0.041 (3)	0.045 (2)	−0.003 (2)	−0.002 (2)	−0.003 (2)
C5	0.045 (3)	0.037 (3)	0.043 (3)	0.003 (2)	−0.005 (2)	−0.001 (2)
C6	0.038 (2)	0.029 (2)	0.037 (2)	−0.0042 (19)	−0.0026 (19)	0.0021 (19)
C7	0.046 (3)	0.028 (2)	0.036 (2)	0.000 (2)	−0.002 (2)	0.0057 (19)
C8	0.048 (3)	0.035 (3)	0.036 (2)	−0.008 (2)	−0.0007 (19)	0.004 (2)
C9	0.041 (3)	0.024 (3)	0.045 (3)	0.000	−0.007 (3)	0.000
C10	0.063 (3)	0.050 (3)	0.066 (3)	−0.022 (3)	−0.006 (3)	−0.002 (3)

### Geometric parameters (Å, º)

**Table:**

I1—C2	2.103 (4)	C4—C5	1.358 (6)
I2—C4	2.092 (4)	C5—C6	1.409 (6)
Cu1—O1	1.902 (3)	C5—H5	0.9300
Cu1—O1^i^	1.902 (3)	C6—C7	1.439 (6)
Cu1—N1^i^	1.944 (3)	C7—H7	0.9300
Cu1—N1	1.944 (3)	C8—C9	1.538 (5)
O1—C1	1.301 (5)	C8—H8A	0.9700
N1—C7	1.283 (5)	C8—H8B	0.9700
N1—C8	1.466 (5)	C9—C10	1.522 (6)
C1—C2	1.414 (6)	C9—C10^i^	1.522 (6)
C1—C6	1.422 (6)	C9—C8^i^	1.538 (5)
C2—C3	1.364 (6)	C10—H10A	0.9600
C3—C4	1.391 (6)	C10—H10B	0.9600
C3—H3	0.9300	C10—H10C	0.9600
			
O1—Cu1—O1^i^	91.04 (17)	C5—C6—C1	120.4 (4)
O1—Cu1—N1^i^	157.69 (13)	C5—C6—C7	116.9 (4)
O1^i^—Cu1—N1^i^	93.51 (13)	C1—C6—C7	122.7 (4)
O1—Cu1—N1	93.51 (13)	N1—C7—C6	125.7 (4)
O1^i^—Cu1—N1	157.69 (13)	N1—C7—H7	117.2
N1^i^—Cu1—N1	90.5 (2)	C6—C7—H7	117.2
C1—O1—Cu1	127.3 (3)	N1—C8—C9	113.4 (3)
C7—N1—C8	119.2 (4)	N1—C8—H8A	108.9
C7—N1—Cu1	125.5 (3)	C9—C8—H8A	108.9
C8—N1—Cu1	115.2 (3)	N1—C8—H8B	108.9
O1—C1—C2	120.0 (4)	C9—C8—H8B	108.9
O1—C1—C6	124.3 (4)	H8A—C8—H8B	107.7
C2—C1—C6	115.7 (4)	C10—C9—C10^i^	109.2 (6)
C3—C2—C1	122.9 (4)	C10—C9—C8	107.8 (3)
C3—C2—I1	119.0 (3)	C10^i^—C9—C8	110.4 (3)
C1—C2—I1	118.1 (3)	C10—C9—C8^i^	110.4 (3)
C2—C3—C4	120.2 (4)	C10^i^—C9—C8^i^	107.8 (3)
C2—C3—H3	119.9	C8—C9—C8^i^	111.2 (5)
C4—C3—H3	119.9	C9—C10—H10A	109.5
C5—C4—C3	119.6 (4)	C9—C10—H10B	109.5
C5—C4—I2	121.1 (3)	H10A—C10—H10B	109.5
C3—C4—I2	119.3 (3)	C9—C10—H10C	109.5
C4—C5—C6	121.3 (4)	H10A—C10—H10C	109.5
C4—C5—H5	119.4	H10B—C10—H10C	109.5
C6—C5—H5	119.4		
			
O1^i^—Cu1—O1—C1	168.3 (4)	C2—C3—C4—I2	178.6 (3)
N1^i^—Cu1—O1—C1	−89.9 (5)	C3—C4—C5—C6	0.1 (7)
N1—Cu1—O1—C1	10.1 (3)	I2—C4—C5—C6	−178.9 (3)
O1—Cu1—N1—C7	−7.0 (4)	C4—C5—C6—C1	1.0 (6)
O1^i^—Cu1—N1—C7	−108.3 (4)	C4—C5—C6—C7	−177.3 (4)
N1^i^—Cu1—N1—C7	151.1 (4)	O1—C1—C6—C5	178.8 (4)
O1—Cu1—N1—C8	168.9 (3)	C2—C1—C6—C5	−1.6 (6)
O1^i^—Cu1—N1—C8	67.5 (5)	O1—C1—C6—C7	−3.0 (6)
N1^i^—Cu1—N1—C8	−33.1 (2)	C2—C1—C6—C7	176.6 (4)
Cu1—O1—C1—C2	173.7 (3)	C8—N1—C7—C6	−175.1 (4)
Cu1—O1—C1—C6	−6.8 (6)	Cu1—N1—C7—C6	0.5 (6)
O1—C1—C2—C3	−179.1 (4)	C5—C6—C7—N1	−175.5 (4)
C6—C1—C2—C3	1.3 (6)	C1—C6—C7—N1	6.2 (7)
O1—C1—C2—I1	2.2 (5)	C7—N1—C8—C9	−111.6 (4)
C6—C1—C2—I1	−177.3 (3)	Cu1—N1—C8—C9	72.3 (4)
C1—C2—C3—C4	−0.4 (6)	N1—C8—C9—C10	−157.0 (4)
I1—C2—C3—C4	178.3 (3)	N1—C8—C9—C10^i^	83.8 (5)
C2—C3—C4—C5	−0.4 (6)	N1—C8—C9—C8^i^	−35.9 (2)

Symmetry code: (i) −*x*, *y*, −*z*+1/2.
